# Early Optical Coherence Tomography Biomarkers for Selected Retinal Diseases—A Review

**DOI:** 10.3390/diagnostics13142444

**Published:** 2023-07-21

**Authors:** Ewa Goździewska, Małgorzata Wichrowska, Jarosław Kocięcki

**Affiliations:** 1 Department of Ophthalmology, Poznan University of Medical Sciences, 60-569 Poznań, Poland; malgorzata.wichrowska@usk.poznan.pl (M.W.); jaroslaw.kociecki@usk.poznan.pl (J.K.); 2 Doctoral School, Poznan University of Medical Sciences, 61-701 Poznań, Poland

**Keywords:** optical coherence tomography, OCT biomarker, age-related macular degeneration, idiopathic macular telangiectasia, chloroquine maculopathy

## Abstract

Optical coherence tomography (OCT) is a non-invasive, easily accessible imaging technique that enables diagnosing several retinal diseases at various stages of development. This review discusses early OCT findings as non-invasive imaging biomarkers for predicting the future development of selected retinal diseases, with emphasis on age-related macular degeneration, macular telangiectasia, and drug-induced maculopathies. Practitioners, by being able to predict the development of many conditions and start treatment at the earliest stage, may thus achieve better treatment outcomes.

## 1. Introduction

The National Institutes of Health defines a biomarker as a quantifiable biological parameter that is measured and evaluated as an indicator of normal biological, pathogenic, or pharmacologic response to a therapeutic intervention [1].

There is currently a high demand for diagnostic biomarkers that can assist specialists in detecting early signs of retinal diseases. There were attempts to find such biomarkers using fluorescein and indocyanine green angiography, but optical coherence tomography (OCT) is a better potential tool that can serve this purpose, as it is a rapid, highly accessible, and non-invasive imaging method [2]. This imaging technique produces high-resolution cross-sectional images of tissue and allows for in vivo imaging with no influence on the scanned tissue [3].

OCT produces images through the use of interferometry to measure the delay and amplitude of reflected or backscattered light. A light beam is directed towards the retina or front part of the eye. Then, the depth is measured by interfering with the light reflected or scattered back from ocular structures with light that has gone along a predetermined reference path [4].

In the beginning, OCT could only achieve an axial resolution in the range of 10 to 20 µm. This level of resolution was insufficient to distinguish between the various structures of the retina. As a result, the neurosensory retina and the retinal pigment epithelium (RPE) appeared as a solitary, strongly reflective band [5]. Time-domain OCTs (TD-OCTs), like Stratus OCT (Carl Zeiss Meditec, Inc., Dublin, CA, USA), were earlier generation types of OCT, which employed a time-domain method that allowed a 400 A-scans per second scan rate. Those machines were able to achieve an axial resolution of approximately 8 to 10 μ [6]. The development of the newer generation SD-OCT, such as the Spectralis (Heidelberg Engineering GmbH, Heidelberg, Germany) and Cirrus OCT (Carl Zeiss Meditec, Dublin, CA, USA), enabled the achievement of an axial resolution of 5 to 7 µm, faster scan rates at 20,000 up to 100,000 A-scans per second, and better signal-to-noise ratio compared with TD-OCT [5,7]. This is due to an interferometer and spectrometer in the newer generation machines, which analyze backscattered light interference patterns simultaneously through the Fourier transform algorithm [8]. The enhanced ability to distinguish between various layers of the retina makes this technique well suited for the clinical diagnosis and treatment of eye conditions.

Moreover, there is a novel method called optical coherence tomography angiography (OCTA)—quite a new variety of OCT that is another possible method for determining diagnostic biomarkers [9]. OCTA enables the visualization of the retinal vessel network and choriocapillaris with high resolution but without intravenous contrast. The signal emitted by structural tissue stays constant, while the signal from red blood cells in flowing blood varies with time. Thus, changes in the OCT signal between successive scans can indicate changes in blood circulation [10]. 

The review aimed to summarize the current literature on OCT biomarkers for retinal diseases such as age-related macular degeneration, idiopathic macular telangiectasia, and chloroquine maculopathy and to discuss the potential clinical implications of using these biomarkers in routine ophthalmic practice.

In our review, we have focused on describing OCT changes in AMD due to its high prevalence and global significance. We aimed to emphasize the role of OCT in diagnosing AMD and highlight the importance of early biomarker identification for early detection at less advanced stages of the disease. Additionally, we provided representative examples from other groups of retinal diseases—drug-induced maculopathies and idiopathic macular telangiectasia. We specifically chose to discuss macular telangiectasia type 2 as it is the most common subtype among idiopathic macular telangiectasia cases. Regarding chloroquine maculopathy, we included it in our review due to its prominence as a notable example of ocular toxicity associated with medication, supported by a wealth of available literature on the topic compared to other drug-related maculopathies.

## 2. Materials and Methods

The articles were reviewed to evaluate the correlation between OCT biomarkers and the early diagnosis or prognosis of certain diseases. Due to significant variations in the methodology of these studies, a meta-analysis was not feasible, and a narrative synthesis was conducted using a best-evidence synthesis approach. A thorough review of the relevant literature was conducted through PubMed, utilizing appropriate keywords to retrieve research articles on early OCT biomarkers in selected retinal diseases.

To be considered for inclusion, studies had to (a) involve patients with retinal diseases such as age-related macular degeneration, idiopathic macular telangiectasia, and drug-induced maculopathies; (b) assess early OCT or OCTA biomarkers; (c) examine the correlation between these biomarkers and prognosis or early diagnosis, and (d) be written in English. Studies were excluded if they (a) did not focus on specific retinal diseases and (b) lacked descriptions of the influence of OCT biomarkers during study monitoring.

## 3. Age-Related Macular Degeneration

Recently, age-related macular degeneration (AMD) has remained a leading cause of visual disability globally [9]. It has been suggested that AMD develops as a result of chronic pathological cycles of inflammation and oxidative stress [11,12].

**Figure 1:**
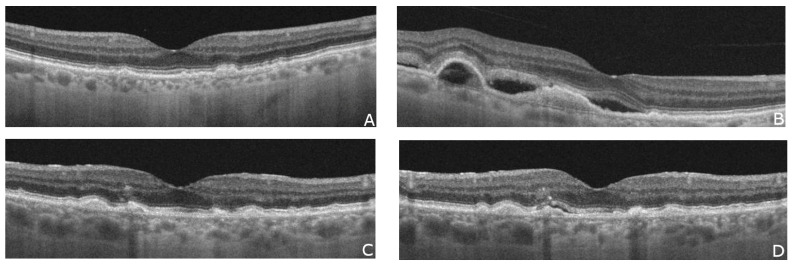
The OCT scans of two patients show examples of fellow eye involvement in AMD after some time from the initial diagnosis of the first eye. In the case of the first patient, the left eye was treated with 19 anti-VEGF injections starting in January 2018. (**A**) shows the macula of the right eye just before the conversion to the wet form of AMD in April 2023 (**B**). The second patient was initially treated with 12 anti-VEGF injections in the left eye starting in April 2020. (**C**) shows an OCT scan of the right eye before the appearance of subretinal fluid in September 2021 (**D**).

Although AMD is a bilateral disease, simultaneous bilateral involvement is relatively rare. The condition typically affects one eye initially and becomes visible in the fellow eye sometime after the initial diagnosis [13] (Figure 1). AMD has been historically categorized into two main subtypes based on the presence of neovascularization, which occurs in about 10–15% of all AMD cases [14]. Consensus on Neovascular AMD Nomenclature (CONAN) was published in the year 2019 and unified the previous classification of neovascularization in AMD [15]. The old term “choroidal neovascularization” (CNV) was replaced with “macular neovascularization” (MNV), as it is known that neovascularization may start in the outer retina. In many older scientific papers, we can still find the term CNV instead of the more appropriate MNV. Nowadays, the term CNV is still used to describe the formation of pathological new blood vessels in diseases other than AMD [16]. In certain parts of this review, we will use the term “CNV” to maintain the original context when discussing neovascularization in neovascular AMD (nAMD), specifically when referring to papers that originally used this term. 

Type 1 MNV is located under the retinal pigment epithelium (RPE) and is associated with pigment epithelial detachment (PED), while type 2 MNV is present above the RPE in the subretinal layer. The initial location of type 3 neovascularization is intraretinal. The terms type 1 MNV and type 2 MNV replaced occult CNV and classic CNV, respectively. Type 3 MNV is used instead of retinal angiomatous proliferation (RAP) [17]. It was estimated that about 30% of patients develop nAMD in the fellow eye 2–6 years after the diagnosis of the neovascular membrane in the initial eye [18]. Rates of nAMD development in unaffected eyes of patients who have had neovascular AMD in their first eye have been established at approximately 12% per year [19,20]. Given these estimates, AMD fellow eyes may have pre-disease characteristics, even if there are no drusen and/or pigment abnormalities in fundus evaluation [13].

The phenotype and course of the disease are unpredictable, and some patients may never reach the advanced stage of the disease. However, others will develop macular neovascularization very early. The reason why the natural history of the disease varies so much between individuals is not fully understood yet. Nevertheless, certain factors such as genetic polymorphisms (e.g., in CFH and ARMS2 genes) and environmental factors (e.g., smoking) have been associated with a higher risk of nAMD [21].

Early diagnosis of the exudative form of AMD is crucial to prevent functional impairment and improve quality of life. It has been shown that better visual outcomes are obtained when treatment is implemented quickly in eyes with better visual acuity at baseline [19,22]. Hence, frequent monitoring of the fellow eye is the key to detecting the possible active neovascular membrane early [23]. There are many studies aimed at discovering early OCT biomarkers to enable earlier detection of changes leading to the development of AMD (Table 1).

### 3.1. Drusen Height

Hilely et al. conducted a study to investigate the presence of subretinal fluid in non-neovascular AMD [24]. They proposed that large drusen causing reduced choroidal perfusion might lead to RPE pump failure, which could explain the development of underlying subretinal fluid. This research suggests that as the height of the drusen increases, and the RPE separates from the choroid, RPE insufficiency may occur and can be detected as early signs of atrophy on OCT imaging.

Based on this report, another study by Au et al. was conducted to correlate increasing drusen height and the distance between the choriocapillaris and RPE with OCT predictive biomarkers of atrophy [25]. This study showed for the first time that drusen height, more so than GHD (greatest horizontal diameter), is correlated with the presence of OCT predictors of atrophy, such as disruption of the external limiting membrane (ELM) and ellipsoid zone (EZ), RPE thickening and thinning, intraretinal hyper-reflective foci (HRF), choroidal transmission, and the presence of hyporeflective cores. The authors suggested that drusen height may also be related to the development of RPE impairment and disruption, which is consistent with earlier studies [37,38]. Drusen height may serve as an early biomarker in the future for targeting early intervention to prevent atrophy and vision loss.

### 3.2. Drusen Volume

An increased volume of drusen has been proposed as a possible risk factor for the development of late-stage AMD [26]. Quantifying drusen volume through OCT could be a promising biomarker for identifying patients at higher risk of disease progression. Abdelfattah et al. evaluated drusen volume in correlation with progression to late AMD in individuals who had evidence of macular neovascularization in the fellow eye and observed that this parameter was the strongest predictor for the onset of advanced AMD. Those patients underwent follow-up at 12 and 24 months [26]. Lamin et al. discovered that the total amount of drusen measured by optical coherence tomography increased before the development of neovascular age-related macular degeneration in the unaffected eyes of individuals with neovascular AMD in one eye [27]. This suggests that monitoring the total drusen volume with OCT could be a useful method for detecting early signs of neovascular AMD in those at risk for disease development. Furthermore, they found that a year before conversion, the mean change in macular drusen load in eyes that converted to wet AMD rose.

### 3.3. RPE Changes

In a retrospective study by Amissah-Arthur et al., the authors analyzed the database of the nAMD treatment program to identify all patients who received bilateral injections of ranibizumab in two years. Their goal was to identify patients who developed nAMD in their second eyes while being treated for unilateral nAMD [19]. The study found that in many cases, there were abnormalities in the RPE without any visible alterations in the outer neural retina in multiple appointments before the diagnosis of second eye conversion. The study suggested that it may be possible to diagnose conversion to a wet form of AMD in the fellow eye by looking for small changes in RPE contours or elevation rather than waiting for sub- or intra-retinal fluid to be visible on OCT images, which is currently the most common practice among specialists.

Macular pigment optical density (MPOD) is an indicator of the levels of macular pigment (MP), which is composed of lutein, zeaxanthin, and vera-zeaxanthin, acting as an antioxidant in the retina [13]. Low dietary intake and low serum levels of lutein can lead to low MPOD levels. It has been shown that, in AMD patients, the MPOD level is lowered, indicating that MPOD could be a biomarker of AMD development [39].

The measurement of photoreceptor outer segment (PROS) length, where the visual pigment is primarily located, can be performed using OCT scans. However, its assessment in AMD has not been extensively studied [13]. The PROS consists of numerous folded plasma membrane discs that contain visual pigments. Therefore, any changes in the PROS could potentially serve as a sensitive biomarker for the degeneration of photoreceptors [40].

In the study by Nagai et al., MPOD and PROS length were measured in the macular region. The researchers then compared the data obtained from AMD fellow eyes to the data from age-matched control eyes of individuals without retinal diseases [13]. The study excluded AMD fellow eyes with drusen and/or pigment abnormality. This was the first study to demonstrate changes in PROS length in AMD fellow eyes. The study showed that MPOD and PROS length were significantly lower in the fellow eyes of AMD patients compared to the eyes of control participants. This was also reported in other studies before for MPOD [39,41]. Some AMD fellow eyes, on the other hand, revealed obvious abnormalities solely in terms of MPOD, while others showed it only in terms of PROS length. The authors suggested that the combination of MPOD and PROS parameters may increase accuracy.

### 3.4. Geographic Atrophy

Only a few clinical trials are focused on preventing the progression of intermediate AMD to geographic atrophy, and there is a need to identify reliable early clinical biomarkers to predict atrophy development. The Classification of Atrophy Meetings (CAM) Report 5 publication provided a comprehensive summary of the features characteristic of geographic atrophy [28].

This publication distinguished two stages of atrophy in AMD, named according to the affected anatomic layers on OCT: cRORA (complete retinal pigment epithelium and outer retinal atrophy) and iRORA (incomplete RPE and outer retinal atrophy). The stage named cRORA is defined by a region of choroidal hypertransmission of 250 mm or more in diameter, a zone of attenuation or disruption of the RPE of 250 mm or more in diameter, and evidence of overlying photoreceptor degeneration, all occurring in the absence of signs of an RPE tear [37]. The term iRORA includes all cases in which OCT signs are present but do not fulfill all of the criteria for cRORA [42].

As mentioned before, the main features defining non-neovascular AMD are called drusen, which are deposits that accumulate between the basal lamina of the RPE and the inner collagenous layer of Bruch’s membrane. Their presence is a risk factor for the development of geographic atrophy as well as macular neovascularization or both [25,27].

OCT predictors of atrophy include intraretinal hyperreflective foci, focal RPE thickening, and choroidal hypertransmission, which are the earliest signs of RPE damage and disturbance that may lead to geographic atrophy [28,43].

### 3.5. Choriocapillaris

According to different studies, loss of the choriocapillaris supposedly occurs in areas with intact RPE in nAMD. Postmortem analysis of the eyes of the patients with clinically documented early AMD showed choriocapillaris dropout with confocal microscopy and an approximately 20% reduction in vessel density in the choriocapillaris of the macular area [44]. These findings indicate that alterations in the choriocapillaris could potentially be detected at an early stage, even before they become visible during a fundus examination. Harada et al. conducted a study to identify early changes in the choriocapillaris in AMD fellow eyes that did not exhibit typical fundoscopy findings, such as drusen or MNV [9]. The authors had previously reported that compared to age-matched healthy individuals, AMD fellow eyes have a shorter photoreceptor outer length and lower macular pigment optical density [13,41].

They evaluated en-face images of the choriocapillaris OCT slab of 24 AMD fellow eyes of 24 patients with unilateral late AMD. To assess choriocapillaris flow deficits in AMD fellow and control eyes, the authors calculated the choriocapillaris flow area (CCFA) ratio by measuring the percentage of the slab area to the analyzed area from OCTA images. The CCFA ratio was then compared between the two groups in four locations around the fovea. The mean CCFA ratio of the macular area was smaller in AMD high-risk fellow eyes with no particular findings such as drusen or MNV in the fundus than in the control eyes [10]. The decreased CCFA ratio observed in the AMD fellow eyes might indicate a decline in choriocapillaris flow, which could serve as an early indicator of AMD development in these eyes, according to the study.

According to various studies, a decline in choroidal vessel density can be observed in the early stages of AMD [45,46]. However, in the early stages of AMD, vascular changes extend beyond the choroidal blood supply. Toto et al. reported the loss of vascular density in the superficial capillary plexus (SCP) and the deep capillary plexus (DCP) in intermediate AMD. In that study, choroidal thickness was also found to be reduced in patients with AMD [47].

Later work by Trinh et al. was also conducted to examine with OCTA the changes in the retinal vasculature and ganglion cell layer (GCL) thickness in intermediate AMD [29]. GCL was examined to determine if there was an association between changes in the vasculature supplying the inner retina. In comparison to control eyes, the authors discovered a substantial reduction in the SCP’s vascular density in AMD patients. However, vascular density in SCP was not significantly decreased. Additionally, they noticed a reduction in GCL thickness in AMD eyes, which suggests that structural loss in the early stages of AMD may be linked to retinal vascular alterations. As different OCTA modalities utilize different scanning techniques and definitions to automatically separate the retinal vasculature from the scan, differences between OCTA studies may be device-related.

### 3.6. Predicting the Type of MNV in the Fellow-Eye

There were several attempts to predict the type of macular neovascularization that may develop in the unaffected eye of patients who have already experienced nAMD in one eye.

In a study by Lamin et al., the authors investigated whether there is any correlation between drusen and retinal layer volume changes before conversion onset and the subsequent choroidal neovascularization type in the same eye [14]. The comparison of drusen load between the two types of CNV revealed a significant rise in the area and volume of drusen during the 12 months preceding the onset of occult CNV. On the other hand, the mean drusen count, area, or volume did not show any significant differences in eyes with classic CNV. In the comparison of classic CNV with occult CNV, the conclusion was different—a greater increase in OCT drusen area and volume was seen in the year before developing occult CNV compared to other eyes that developed classic CNV. They also compared retinal layer volumes between the two CNV types. The study found a decrease in outer nuclear layer (ONL) volume and an increase in outer plexiform layer volume in eyes that developed occult CNV. Furthermore, the rate of increase in drusen load and reduction in ONL were significant features observed in eyes developing occult CNV. The study also noted a high level of symmetry in CNV types between the two eyes, suggesting that it may be possible to predict the CNV type that will most likely develop in a fellow eye of a patient with unilateral wet AMD. Prior studies have indicated that individuals diagnosed with unilateral occult CNV are at an elevated risk of experiencing the development of occult CNV in their fellow eye [48]. In eyes with nonexudative AMD, there may exist type 1 neovascular membranes that are asymptomatic and inactive and do not display exudative changes yet. These lesions have unique features that can be observed on B-scan SD-OCT and can also be identified with en-face OCT.

In a study by Mentes et al., B-scan OCT images of all examined eyes showed RPE elevations and irregularities caused by moderately reflective material in the sub-RPE space without IRF/SRF accumulation [30]. A total of 88.8% of eyes showed sub-RPE hyperreflective lesions consistent with type 1 neovascularization on en-face OCT images. In this study, the authors found that type 1 neovascularization showed moderate reflectivity and caused the RPE to appear slightly elevated, irregular, and undulating, leading to the creation of the “double-layer sign” (DLS). The DLS describes the shallow and irregular elevation of the retinal pigment epithelium from the underlying Bruch’s membrane visualized on the OCT [49]. Various clinical studies have shown before that quiescent neovascular lesions not yet causing exudative findings may be present in eyes with not only nonexudative AMD but also polypoidal choroidal vasculopathy (PCV) and angioid streaks [50].

Shi et al. tried to determine if the DLS predicted subclinical macular neovascularization [31]. Two junior graders independently examined the scans and subsequently arrived at a consensus regarding the grading. Remarkably, all graders observed statistically significant associations between type 1 MNV and the presence of the double-layer sign. They concluded that the DLS can be used to identify type 1 MNV with good predictive values in eyes with nonexudative AMD.

De Oliveira Dias et al. investigated the presence of subclinical MNV in patients with neovascular AMD in one eye and nonexudative AMD in the fellow eye [32]. They examined 160 patients with intermediate AMD or geographic atrophy secondary to nonexudative AMD in one eye and exudative AMD in the fellow eye. They detected type 1 neovascularization and type 3 subclinical neovascular membranes in 14.4% of the eyes and found that all type 1 neovascular lesions caused RPE elevation in OCT. Three eyes with RPE elevations in OCT developed exudation eight weeks later. Their conclusion was that, within a 12-month timeframe, eyes exhibiting documented subclinical MNV demonstrated a higher risk of exudation in comparison to eyes without detectable MNV. Nowadays, the measurement of CVI is performed with semi-automated software [51]. However, the availability of an automated CVI analysis tool could prove beneficial. In such a scenario, reduced CVI values observed in the unaffected eye could serve as a valuable OCT biomarker for assessing the likelihood of neovascularization and assist in developing an appropriate follow-up strategy for individuals with type 3 neovascularization in one eye.

In the study by Nassisi et al., the authors aimed to confirm that four previously reported OCT risk factors, intraretinal hyperreflective foci (IHRF), hyporeflective foci (hRF) within drusenoid lesions (DLs), subretinal drusenoid deposits (SDD), and higher drusen volume, were associated with progression to late AMD in the fellow eyes of patients newly diagnosed with MNV [33]. They found that the presence of intraretinal IHRF, hRF within a drusen-like lesion, and SDD, along with drusen volume ≥0.03 mm within the central 3 mm circle, were significantly associated with an increased risk of progression to late age-related macular degeneration. The strongest individual predictor for progression to late AMD (both atrophy and MNV) was IHRF [52]. In their previous work, they showed that IHRF commonly precedes the development of type 3 MNV (retinal angiomatous proliferation) [53]. Subretinal drusenoid deposits (or reticular pseudodrusen) were determined to be a consistent risk factor for progression to both atrophy and MNV by the previous literature in our analysis [54,55]. Overall, drusen volume was the least predictive of the four parameters and also the only one insignificantly associated with the development of MNV.

On OCT imaging, subretinal drusenoid deposits (SDDs) appear as hyper-reflective interlacing drusen-like deposits located above the retinal pigment epithelium (RPE) in the outer macular region, commonly in the superior or superotemporal area. There is a strong association between the presence of SDDs and the development of geographic atrophy (GA) and neovascular AMD, specifically type 3 macular neovascularization, according to reports [54,56,57].

Kang et al. investigated the chorioretinal thickness and RPE degenerative features of eyes with AMD and SDDs according to the presence of MNV in the fellow eyes [34]. Several studies suggested that early AMD eyes with SDDs are prone to overall chorioretinal degeneration, including of the RPE [58]. Fellow eyes with neovascular AMD showed greater proportions of RPE degeneration and a thicker retina and choroid. 

It was found that eyes with RPE degeneration had lower thickness measurements in both the retina and choroid when compared to eyes without RPE degeneration [58].

Retinal angiomatous proliferation (RAP) is a type of neovascular age-related macular degeneration, also classified as type 3 neovascularization [59]. The term “retinal angiomatous proliferation” was first proposed by Yanuzzi et al. in 2001. In this type of nAMD, neovascular membranes originate from the proliferation of intraretinal capillaries, which is related to a telangiectatic response [60]. The most characteristic feature of RAP is the intraretinal location of the neovessels. It was suggested that type 3 neovascularization tends to have bilateral involvement and a poor prognosis [61]. In a study by Kwak et al., the authors analyzed the incidence of type 3 neovascularization in the unaffected fellow eye and various risk factors that may lead to the development of fellow eye neovascularization [35]. The study found a significant correlation between the incidence of fellow eye neovascularization and the presence of large soft drusen, reticular pseudodrusen, and lower choroidal vascularity index (CVI) values. There is a possibility that low CVI values in the unaffected fellow eyes of patients with RAP may be associated with possible subclinical disease. Nowadays, the CVI is measured manually, but if an automated CVI analysis tool becomes available, lower CVI values in the fellow eye could be considered a useful OCT biomarker for evaluating the risk for neovascularization and may help in establishing a proper follow-up plan for patients with type 3 neovascularization in one eye.

Toprak et al. conducted a study to analyze the impact of soft drusen on the reflectivity of the retinal pigment epithelium (RPE), ellipsoid zone (EZ), and external limiting membrane (ELM) in the early stages using OCT image analysis [36]. Their research comprised patients with non-neovascular AMD (with intact RPE, EZ, and ELM bands on OCT) and age- and sex-matched healthy controls with normal OCT. Then, OCT image analysis was performed by a single masked physician. The reflectivities of RPE, EZ, and ELM, the number of drusen, and drusen characteristics were evaluated based on the macular OCT scan. In the non-neovascular AMD group, absolute EZ and RPE reflectivities were significantly lower compared to those of the control eyes, suggesting early photoreceptor damage before the disruption of these layers on OCT scans. 

Schick et al. conducted a study to identify potential indicators of early or late onset of advanced AMD by examining the morphologic characteristics of the fellow non-affected eye in patients with unilateral neovascular AMD in its early stages and comparing them to patients with either new-onset unilateral CNV or non-neovascular AMD [21]. The research suggests that the occurrence of more than twenty macular drusen is more prevalent in the non-affected eyes of individuals with early onset of unilateral neovascular AMD, indicating a potential biomarker for a more severe progression of the disease. RPE atrophy, as well as reticular pseudodrusen (RPD), were more frequently present in AMD patients over 80 years old compared to patients with an early onset of neovascular AMD. Retinal pigment epithelium atrophy was more frequently present in elderly patients in general. The study found that the prevalence of RPD was higher in both elderly groups compared to the early-onset CNV group, indicating that the likelihood of developing RPD increases with age. Moreover, RPDs were more commonly observed in the late-onset CNV group than the no-CNV group, which is consistent with previous research indicating a link between RPD and neovascular AMD. Hyperreflective dots (HRD) were more frequently detected in the late-onset CNV group compared to the no-CNV group. The main conclusions of that study were that patients with early onset of neovascular AMD showed a higher prevalence of a large number of macular drusen in the non-affected eye, while HRD and RPD were more frequently observed in elderly patients with neovascular AMD. Furthermore, the detection of retinal pigment epithelium atrophy was more common in older AMD patients regardless of the presence of CNV.

## 4. Idiopathic Macular Telangiectasia

Idiopathic macular telangiectasia, also known as IMT or MacTel, was initially identified by Gass and Oyakawa as a condition involving telangiectatic incompetent capillaries that provide blood supply to the area surrounding the fovea [62]. MacTel type 1 is a unilateral developmental condition and is usually identified by the presence of aneurysms and telangiectasia on the temporal side of the macula, along with exudation and cystoid macular edema (CME). With a preference for women and an average patient age over 40, MacTel type 2 is an acquired syndrome that is frequently bilateral and asymmetric. It affects the macular Müller cells and capillary network and is associated with changes in the inner and outer retinal structure, often leading to the development of abnormal neovascular complexes [63,64]. The third type of MacTel is an acquired, bilateral, and occlusive macular telangiectasia that causes progressive vision loss. Although this type has not been thoroughly understood, it has been observed that all documented cases have been linked to a systemic vascular or cerebral disease [65].

In 1993, Gass and Blodi developed a clinical classification system for type 2 MacTel based on clinical and angiographic findings, dividing it into five stages [66]. A classification system comprising five clinical stages has been proposed, utilizing biomicroscopy and fluorescein angiography findings as the basis for categorization. Stage 1 changes in macular telangiectasia include the presence of diffuse hyperfluorescence, which primarily affects the temporal parafoveal area of the retina. As the disease progresses to stage 2, a lack of transparency is visible upon examination through a microscope, while angiography shows mild capillary telangiectasia. Stage 3 is characterized by the presence of dilated right-angle venules and expanded capillaries, while stage 4 is marked by intraretinal pigment migration. In stage 5, neovascularization, hemorrhage, and exudation are common characteristics. Yannuzzi et al. simplified the classification by Gass and Bodi and suggested two distinct stages: non-proliferative and proliferative [67]. Similar to AMD, various efforts were made to identify early OCT biomarkers to anticipate the onset and predict the progression of idiopathic macular telangiectasia, especially type 2 (Table 2).

The earliest sign noted on OCT scans is an enlargement of the foveal pit in the temporal region caused by thinning of the temporal juxtafoveal retina. The majority of thinning takes place in the outer nuclear/Henle’s fiber layer [63].

**Figure 2:**
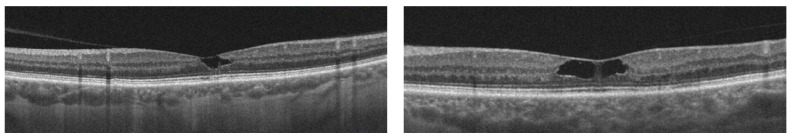
The OCT scans of two different patients show examples of inner retinal hypo-reflective cavities in macular telangiectasia type 2.

Some studies suggest that the most frequently observed OCT features in patients with macular telangiectasia type 2 include hypo-reflective cavities in both the inner and outer neurosensory retina, as well as disruptions in the external limiting membrane (ELM), photoreceptor inner segment–outer segment border, and interdigitation zone [68] (Figure 2).

On OCT, early subretinal neovascularization may be indicated by a thicker temporal retina compared to the nasal fovea without any retinal fluid. Thick, hyper-reflective lesions in the outer retina and highly reflective dots in the inner and outer nuclear layers may also be visible [69]. The deep capillary network located temporal to the fovea is the primary localization of the vascular changes in type 2 MacTel. These changes are characterized by a decrease in vascular density and the presence of telangiectatic vessels, which can be visualized using OCT angiography. As the disease progresses, the capillary network around the fovea is affected in both the superficial and deep plexus [70].

A large cohort of type 2 MacTel patients was included in a retrospective observational analysis by Venkatesh et al. The authors characterized the OCT imaging findings and their association with the various clinical stages of fundus examination [65]. They further established a connection between the OCT findings and MacTel type 2 pathogenesis. 

According to previous research, the hyperreflective middle retinal layer (MRL) is considered to be the most frequent early sign in type 2 MacTel. It was found that in type 2 MacTel, there is a loss of Müller cells in the perifoveal region [72]. This loss of those cells is thought to contribute to the increased hyperreflectivity of the MRL, possibly due to decreased structural stability and disruption of the inner blood–retinal barrier [74]. Additionally, increased capillary leakage may also contribute to changes in the reflectivity of the MRL [75].

The visibility of the deep retinal capillaries on OCT is increased due to the loss of Muller cells, resulting in the formation of hyperreflective lesions in the MRL. In a study by Venkatesh et al., the hyperreflective MRL in the perifoveal region (87%) was noted as the most common OCT finding [71].

It is important to distinguish the loss of perifoveal Müller cells from disorganized retinal inner layers in the early stages of the disease. The latter is characterized by a loss of stratification in the IRL. The retinal layer stratification in the MRL seems to be maintained in type 2 MacTel. This supports the hypothesis that hyperreflective MRL is an early sign seen on OCT in type 2 MacTel. As the disease progresses into its proliferative stage, the hyperreflectivity in the affected area may decrease. This could be due to the loss of supporting structures, atrophy in the surrounding tissue, and the formation of new blood vessels in the retina or in the subretinal region.

Another characteristic finding of type 2 MacTel is superficial intraretinal crystals. While the presence of those lesions is useful for early disease diagnosis, they can still be present in all stages of the disease. The shape and form of the superficial intraretinal cells provide additional evidence of Müller cell involvement in the development of type 2 MacTel [75]. In the study by Venkatesh et al., only 3% of cases showed retinal crystals [71].

Retinal pigment clumps (RPC) are described as hyperreflective clumps in the middle or superficial retinal layers with the presence of underlying shadowing. In the study by Venkatesh et al., hyperreflective intraretinal lesions were often linked to pigment plaques, which were observed in 35% of cases. Pigment plaques were more common in the proliferative group (52%). In more than 50% of proliferative cases in the study, neovascularization occurred simultaneously with the retinal pigment clumps. Therefore, the presence of RPC could potentially serve as an early indicator or biomarker for predicting the onset of the proliferative stage of the disease [71].

Another sign that may represent an early biomarker of the neurodegenerative process in MacTel development is clustered hyperreflective foci (HF) at the foveola [76].

Clusters of HF are commonly found in the regions of the foveal avascular zone, where retinal vessels are absent. These foci are often seen around the border of the hyporeflective cavity or distributed vertically from the inner retina to the external limiting membrane (ELM). Clusters of HF have been observed in several retinal diseases, including diabetic retinopathy, age-related macular degeneration, and retinal vein occlusion [77,78,79]. The origin of HF is not entirely clear, although some studies suggest they may be microglia. Additional research is needed, including histopathological and longitudinal studies, to better understand the nature of these HF clusters and their role in the disease progression.

There have been attempts to identify early biomarkers of MacTel development in fellow eyes. In a multicentered case–control study, Alex et al. compared features of the fellow eyes of patients with asymmetric clinical presentation of MacTel to matched controls [80]. In 92.8% of the unaffected MacTel eyes, they showed the presence of hyperreflective outer retinal dots. Another feature was temporal retinal thinning. According to their findings, the earliest indications of MacTel may include temporal retinal thinning and the presence of hyperreflective outer retinal dots in the unaffected eye.

## 5. Drug-Induced Maculopathies

Although some ocular and systemic drugs have therapeutic benefits, they can also cause damage to the retina since they are carried to the retina through the vascular supply. The retina is particularly prone to the effects of these drugs due to its unique dual blood supply from the retinal and choroidal vasculature. This part of the eye can be adversely affected by a variety of substances, for example, quinolines, phenothiazines, and antiretroviral drugs, among others. While cessation of the medication typically leads to reversible toxicity, it is essential to note that some cases may experience permanent or progressive vision loss [81].

OCT is a highly valuable diagnostic tool for ophthalmologists to evaluate and closely monitor any adverse drug effects. The implementation of advanced retinal imaging tools has resulted in the development of strategies to decrease the likelihood of toxicity.

The utilization of OCT biomarkers plays a crucial role in the implementation of these strategies, particularly in the early detection of toxicity (Table 3).

Antimalarial agents are a flagship example of ocular toxicity associated with drugs.

Since its initial description by Cambiaggi in 1957 [85], this matter has been thoroughly examined through extensive research. Chloroquine and hydroxychloroquine are primarily used to treat and prevent malaria and are also recommended for treating connective tissue disorders, autoimmune diseases such as rheumatoid arthritis or systemic lupus erythematosus, and some dermatological and inflammatory diseases [86]. The reason for the visual impairment caused by chloroquine toxicity is thought to be associated with alterations in the RPE, ultimately resulting in the loss of photoreceptors. However, various animal studies have indicated that the initial retinal damage occurs in ganglion cells and that other layers of the retina are affected only at a later stage [75]. Chloroquine or hydroxychloroquine retinopathy is identified through clinical examination by the presence of bilateral pigmentary changes in the macula due to atrophy of the RPE, with the foveal area being spared.

This condition is commonly known as “bull’s eye” maculopathy, characterized by a depigmented ring surrounding the fovea, followed by a ring of hyperpigmentation [86]. In some cases, retinal toxicity can primarily affect the peripheral retina while sparing the macular area. Atrophy of RPE and the neurosensory retina occurs with the progression of retinopathy, and it spreads from the center to the periphery and eventually affects the entire retina [87].

The term “early chloroquine retinopathy” is used to describe the identification of a paracentral scotoma detected through threshold visual field testing, even in the absence of discernible abnormalities in the retina. On the other hand, “advanced retinopathy” is characterized by the presence of parafoveal RPE atrophy [87]. Central visual field analysis and OCT are regarded as the most efficient diagnostic methods for the early detection of hydroxychloroquine maculopathy before significant damage to the photoreceptors occurs [83,88].

Božinovic et al. conducted a study using OCT to assess the macular retinal thickness, including the central foveal thickness, in adult patients with rheumatoid arthritis (RA) who were undergoing chloroquine therapy. The study focused on both the parafoveal and perifoveal regions of the macula. According to their study, OCT imaging revealed localized thinning of retinal layers in the parafoveal region and confirmed the early stage of toxicity before significant visual field loss was detected. These results are consistent with previous findings, which say that changes in retinal thickness and loss of outer retinal layers can be detected using OCT in patients with early-stage chloroquine maculopathy, even when the fundus and visual field tests appear normal [82].

Melles et al. conducted a study and found that in patients undergoing long-term hydroxychloroquine therapy, retinal thickness remains relatively stable over time. However, there may be a critical point after which the retina starts thinning quickly. By utilizing this approach, patients and prescribing physicians can be notified about possible retinal damage. The OCT measurements are widely available and can potentially be automated by manufacturers for early hydroxychloroquine retinopathy diagnosis [89].

**Figure 3:**
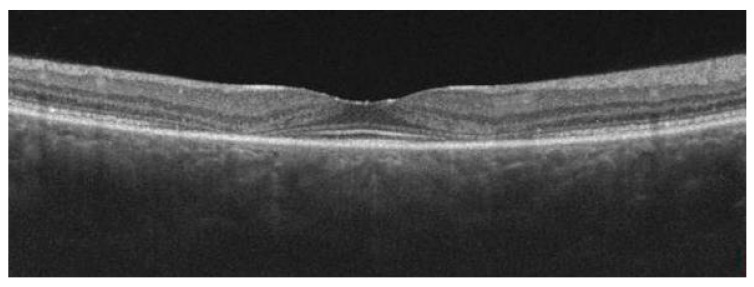
The OCT scan shows the “flying saucer” sign in the early stage of hydroxychloroquine retinopathy.

The initial stage of retinal toxicity is characterized as a focal area of parafoveal inner segment ellipsoid attenuation with subsequent loss, particularly in the inferotemporal quadrant. This can progress to the distinct “flying saucer” sign visible on the spectral domain OCT [83] (Figure 3). The “flying saucer” sign is not pathognomonic nor even necessary for the diagnosis of hydroxychloroquine retinopathy. Moreover, the “flying saucer” sign may not represent the earliest stage of HCQ toxicity visible on SD-OCT.

Stepien et al. described a “preclinical” stage of HCQ toxicity where the photoreceptor inner/outer segments junction appears “moth-eaten” due to preferential loss of cone photoreceptors [84].

## 6. Conclusions

Detecting subclinical signs before AMD changes become visible in fundus examination could be valuable in understanding the fundamental pathogenesis of the disease and developing a screening method for AMD in the early stages [10].

Drusen height can potentially be used as an early biomarker for detecting RPE insufficiency and signs of atrophy on OCT imaging, which can occur as the height of the drusen increases and RPE separates from the choroid [24,25].

Another parameter that can be considered as a possible OCT biomarker for identifying patients at higher risk of disease progression in AMD is drusen volume measured with OCT. An increase in this parameter can occur before the development of neovascular AMD [24,25]. The presence of drusen is considered to be a documented risk factor in the development of geographic atrophy [21,25]. The use of OCT imaging has enabled the detection of subtle changes in the retina, such as abnormalities in RPE contours or elevation, MPOD levels, and PROS length, that may serve as early indicators of disease development and progression [39,40,41].

Several OCT predictors of atrophy, including intraretinal hyperreflective foci, focal RPE thickening, and choroidal hypertransmission, have been identified. Identifying reliable early clinical biomarkers for predicting atrophy development is essential for designing effective prevention strategies, and the OCT features described above may serve as potential biomarkers for predicting atrophy development in intermediate AMD [28,43].

A decrease in choriocapillaris flow was observed in neovascular AMD fellow eyes without MNV, along with the heterogeneous and imbalanced flow. These changes may indicate an early stage of neovascular AMD. More research on choriocapillaris is needed to understand the pathogenesis of AMD [10].

The presence of subclinical neovascularization in patients with nonexudative AMD can be detected using OCT imaging and specifically the double-layer sign (DLS) [31]. Intraretinal hyperreflective foci, hyporeflective foci within drusenoid lesions, and subretinal drusenoid deposits are significantly associated with an increased risk of progression to the late stage of the disease [33,52]. Of these, IHRF is the strongest individual predictor for progression to late AMD [52]. Additionally, SDDs have a strong association with the development of geographic atrophy and type 3 macular neovascularization [54,56,57]. The early detection and monitoring of SDDs and RPE degeneration may be important in preventing the development of neovascular AMD and its associated complications. Risk factors for fellow eye neovascularization in patients with RAP include large soft drusen, reticular pseudodrusen, and lower choroidal vascularity index (CVI) values. Lower CVI values in the unaffected fellow eye may be an OCT biomarker for evaluating the risk for neovascularization and could aid in establishing a proper follow-up plan for patients with RAP [35,60,61].

OCT imaging is a valuable tool in identifying early biomarkers and monitoring the progression of idiopathic macular telangiectasia, especially type 2. The most frequently observed OCT features in type 2 MacTel include hypo-reflective cavities, disruptions in the ELM and photoreceptor layers, and changes in the deep retinal capillaries [61]. The hyperreflective middle retinal layer has been identified as the most common early OCT finding, which may be related to the loss of Müller cells and increased capillary leakage [71]. Additionally, the presence of RPC and clustered HF may be indicative of the proliferative stage and neurodegenerative process, respectively [71,73]. Furthermore, hyperreflective outer retinal dots and temporal retinal thinning in the fellow eye may be early indicators of MacTel [80].

The effectiveness of OCT as a valuable tool in detecting retinal toxicity in patients undergoing chloroquine or hydroxychloroquine therapy has been well established. OCT imaging can reveal localized thinning of retinal layers in the parafoveal region, confirming the early stage of toxicity before significant visual field loss is detected [82]. Additionally, studies have shown that in patients undergoing long-term hydroxychloroquine therapy, retinal thickness remains stable over time, but there may be a critical point after which the retina starts thinning quickly, indicating the need for regular monitoring using OCT [89]. The initial stage of retinal toxicity is characterized by a focal area of parafoveal inner segment ellipsoid attenuation, which can progress to the distinct “flying saucer” sign visible on the spectral domain OCT [83]. However, it is important to note that the “flying saucer” sign is not necessary for the diagnosis of hydroxychloroquine retinopathy [85].

It is important to highlight that, similar to any other imaging method, OCT imaging also has certain limitations. Firstly, OCT images can be affected by artifacts, such as motion artifacts and poor patient fixation, which can reduce image clarity and reliability, potentially impacting diagnostic accuracy [90].

Another significant challenge in OCT imaging is the presence of media opacities. Media opacities refer to conditions where the transparency of ocular media, including the cornea, lens, or vitreous, is compromised. These opacities can include cataracts, corneal scars, vitreous hemorrhages, or other conditions that obstruct or scatter light passing through the eye. They have multiple effects on the quality and clarity of OCT images. Firstly, they attenuate the OCT signal as it passes through the affected media, resulting in decreased signal strength reaching the retina and poorer image quality with reduced visibility of retinal structures. Moreover, opacities can introduce artifacts and distortions in OCT images, such as shadowing, signal attenuation, or the appearance of false structures, which can hinder accurate interpretation and analysis of retinal layers. In severe cases, media opacities can completely block the OCT beam, making it extremely challenging or even impossible to visualize retinal structures. However, the utilization of a longer wavelength light source in SS-OCT, compared to SD-OCT, has the potential to enhance image quality in eyes with media opacity, making SS-OCT a valuable tool for assessing retinal diseases, especially in cases with severe media opacity [91].

Despite the objective nature of OCT measurements, the interpretation of results remains subjective due to the lack of standardized software providing detailed analysis of retinal structure reflectivity. Therefore, the expertise and experience of the examiner play a crucial role in interpreting OCT images [92].

Optical coherence tomography is a readily available and non-invasive imaging method that allows for the detection of various retinal diseases at different stages of development. With the ability to predict the occurrence of many conditions, practitioners can react faster to the preventive or therapeutic needs of each patient.

## Figures and Tables

**Table 1:** Studies on early optical coherence tomography biomarkers linked with age-related macular degeneration (continuation of the table on pages 5–7).

Author	Year	Number of Patients/Eyes Included	Biomarker	Characteristics of Biomarker	Description	Correlation with AMD	Method	The Role of Biomarker in Clinical Practice
Hilely et al. [24]	2021	45 eyes	Drusen height	Quantitative biomarker	The height of the drusen causing reduced choroidal perfusion might lead to RPE ^1^ pump failure	An early sign of atrophy on OCT ^2^ imaging	OCT ^2^	Predictor
Au et al. [25]	2022	155 eyes	Drusen height	Quantitative biomarker	Drusen height, more so than drusen GHD ^3^, is correlated with the presence of OCT ^1^ predictors of atrophy	Early biomarker for targeting early intervention to prevent atrophy and vision loss	OCT ^2^	Predictor
Abdelfattah et al. [26]	2016	89 patients	Drusen volume	Quantitative biomarker	Quantifying drusen volume through OCT ^2^ could be a promising biomarker for identifying patients at higher risk of developing MNV ^4^	Important predictor for the development of advanced AMD ^5^ at 12 and 24 months of follow-up in the fellow eye	OCT ^2^	Predictor
Lamin et al. [27]	2019	248 patients	Drusen volume	Quantitative biomarker	Increase in the overall amount of drusen measured through optical coherence tomography before neovascular AMD ^5^ development	A useful method for detecting early signs of neovascular AMD ^5^ in those at risk for the disease	OCT ^2^	Predictor
Amissah-Arthur et al. [19]	2012	749 patients	RPE ^1^ changes	Qualitative biomarker	Abnormalities in the RPE ^1^ without any visible alterations in the outer neural retina	Possible to diagnose conversion to a wet form of AMD ^5^ in the fellow eye by looking for small changes in RPE ^1^ contours/elevation	OCT ^2^	Predictor
Nagai et al. [13]	2020	30 eyes	PROS ^6^	Quantitative biomarker	The length of the PROS ^6^ where the visual pigment is concentrated	Potential sensitive biomarker for the degeneration of photoreceptors	OCT ^2^	Risk factor
Jaffe et al. (CAM Report 5) [28]	2021	Not available	Intraretinal hyperreflective foci, focal RPE ^1^ thickening, and choroidal hypertransmission	Qualitative biomarker	Early signs of RPE ^1^ damage and disturbance that may lead to geographic atrophy	Predictors of atrophy	OCT ^2^	Predictor
Harada et al. [10]	2022	24 eyes	CCFA ^7^ ratio	Quantitative biomarker	Percentage of choriocapillaris slab area to analyzed area from OCTA ^8^ images	Smaller CCFA ^7^ ratio in AMD high-risk fellow eyes than in control eyes, indicating decline in choriocapillaris flow	OCTA ^8^	Risk factor
Trinh et al. [29]	2019	63 eyes	GCL ^9^ thickness	Quantitative biomarker	Reduction in GCL ^9^ thickness in AMD eyes	Structural loss in the early stages of AMD may be linked to retinal vascular alterations	OCT ^2^/OCTA ^8^	Predictor
Trinh et al. [29]	2019	63 eyes	Vascular density in the superficial capillary plexus (SCP) ^10^	Quantitative biomarker	As different OCTA ^8^ modalities utilize different scanning techniques and definitions to automatically separate the retinal vasculature from the scan, differences between OCTA ^8^ studies may be device-related	Substantial reduction in the SCP’s ^10^ vascular density in AMD patients; however, vascular density in DCP ^11^ was not significantly decreased	OCTA ^8^	Risk factor
Lamin et al. [14]	2020	51 eyes	Drusen load	Quantitative biomarker	Comparison of drusen load between the two types of CNV ^12^	Significant rise in the area and volume of drusen during the 12 months preceding the onset of occult CNV ^12^	OCT ^2^	Risk factor
Lamin et al. [14]	2020	51 eyes	Retinal layer volumes	Quantitative biomarker	Comparison of retinal layer volumes between CNV ^12^ types	Decrease in ONL ^13^ volume and increase in outer plexiform layer volume in eyes with occult CNV ^12^	OCT ^2^	Risk factor
Menteş et al. [30]	2019	27 eyes	Drusen load	Quantitative biomarker	Comparison of drusen load between the two types of CNV ^12^	Significant rise in the area and volume of drusen during the 12 months preceding the onset of occult CNV ^12^	OCT ^2^	Risk factor
Shi et al. [31]	2019	100 eyes	DLS ^14^	Qualitative biomarker	Identification of type 1 MNV in eyes with nonexudative AMD	DLS ^14^ has good predictive values for identifying type 1 MNV ^2^	OCT ^2^	Predictor
Oliveira Dias et al. [32]	2018	160 patients	Subclinical neovascularization	Qualitative biomarker	Neovascularization was not visible on clinical exam, but detected on OCT ^2^ imaging	Increased risk of exudation in eyes with subclinical MNV ^4^ compared to eyes without detectable MNV ^4^	OCTA ^8^	Risk factor
Nassisi et al. [33]	2019	501 eyes	IHRF ^15^, hRF ^16^, DLs ^14^, SDD ^17^, and Drusen volume	Qualitative biomarkers (except drusen volume—quantitative biomarker)	Various features visible on OCT imaging	IHRF ^15^, hRF ^16^ within a drusen-like lesion, and SDD ^17^ were significantly associated with an increased risk of progression to late AMD ^5^. SDD ^17^ is strongly associated with the development of GA ^18^ and type 3 MNV ^4^. Drusen volume was not significantly associated with the development of MNV ^4^	OCT ^2^	Risk factors
Kang et al. [34]	2022	70 eyes	Chorioretinal thickness, RPE ^1^ degenerative features, and SDDs ^17^	Chorioretinal thickness—quantitative biomarker; RPE ^1^ degenerative features and SDDs ^17^—qualitative biomarkers	The thickness of the retina and choroid in eyes with RPE ^1^ degeneration is comparatively reduced to those without this degeneration	Early AMD ^5^ eyes with SDDs ^17^ are prone to overall chorioretinal degeneration. Fellow eyes with neovascular AMD ^5^ showed greater proportions of RPE ^1^ degeneration and a thicker retina and choroid	OCT ^2^	Risk factor
Kwak et al. [35]	2021	93 eyes	CVI ^19^	Quantitative biomarker	Lower CVI ^19^ values in unaffected fellow eyes may be associated with possible subclinical disease	Significant correlation between the incidence of fellow eye type 3 neovascularization and the presence of large soft drusen, reticular pseudodrusen, and lower choroidal vascularity index CVI ^19^ values	OCT ^2^	Risk factor
Toprak et al. [36]	2017	47 eyes	RPE ^1^ and EZ ^20^ reflectivity	Qualitative biomarker	Lower reflectivities suggest early photoreceptor damage	Non-neovascular AMD patients have significantly lower reflectivities compared to healthy controls.	OCT ^2^	Predictor
Schick et al. [21]	2015	104 patients	Macular drusen	Quantitative biomarker	More than 20 drusen in non-affected eyes indicate potential biomarkers for more severe progression of the disease	Prevalence of more than 20 macular drusen is more prevalent in non-affected eyes of individuals with early onset of unilateral neovascular AMD ^5^	OCT ^2^	Risk factor
			RPD ^21^	Qualitative biomarker	Prevalence increases with age and is more common in the late-onset CNV ^12^ group; link with neovascular AMD ^5^	RPDs ^21^ were more commonly observed in the late-onset CNV ^12^ group than in the no-CNV ^12^ group.		Predictor

^1^ RPE—retinal pigment epithelium; ^2^ OCT—optical coherence tomography; ^3^ GHD—greatest horizontal diameter; ^4^ MNV—macular neovascularization; ^5^ AMD—age-related macular degeneration; ^6^ PROS—photoreceptor outer segment; ^7^ CCFA—choriocapillaris flow area; ^8^ OCTA—optical coherence tomography angiography; ^9^ GCL—ganglion cell layer; ^10^ SCP—superficial capillary plexus; ^11^ DCP deep capillary plexus; ^12^ CNV—choroidal neovascularization; ^13^ ONL—outer nuclear layer; ^14^ DLs—drusenoid lesions; ^15^ IHRF—intraretinal hyperreflective foci; ^16^ hRF—hyporeflective foci; ^17^ SDDs—subretinal drusenoid deposits; ^18^ GA—geographic atrophy; ^19^ CVI—choroidal vascularity index; ^20^ EZ—ellipsoid zone; and ^21^ RPD—reticular pseudodrusen.

**Table 2:** The early optical coherence tomography (OCT) findings in idiopathic macular telangiectasia type 2 (MacTel type 2).

OCT Features	Description
Enlarged foveal pit	A thin temporal juxtafoveal retina leads to enlargement of the foveal pit in the temporal region (thinning takes place in the outer nuclear/Henle’s fiber layer) [63].
Hypo-reflective cavities	Located in both inner and outer neurosensory retina [68].
Disruptions of retinal layers	Disruption of the external limiting membrane (ELM), photoreceptor inner segment–outer segment border, and interdigitation zone—one of the most frequently observed OCT features in patients with idiopathic macular telangiectasia type 2 [68].
Thicker temporal retina	Early subretinal neovascularization may be indicated by a thicker temporal retina compared to nasal fovea without retinal fluid [69].
Hyper-reflective lesions	Thick, hyper-reflective lesions in the outer retina, with highly reflective dots in the inner and the outer nuclear layers [69].
Decrease in vascular density	In type 2 MacTel, the earliest vascular changes are observed in the deep vascular plexus, which are characterized by a decrease in vascular density and the presence of telangiectatic vessels (changes can be visualized using OCT angiography) [70].
Hyperreflective middle retinal layer (MRL)	Loss of Müller cells in the perifoveal region may contribute to increased hyperreflectivity of MRL [70,71,72]. In type 2 MacTel, hyperreflective MRL in the perfoveal region was recognized as the most frequent early OCT finding [71].
Superficial intraretinal crystals	Lesions present in all stages of disease provide evidence of Müller cell involvement. Useful for early disease diagnosis [71].
Retinal pigment clumps (RPC)	The presence of retinal pigment clumps can potentially serve as an early indicator or biomarker for predicting the onset of the proliferative stage of the disease [71].
Clustered hyperreflective foci (HF)	Clustered hyperreflective foci are the early biomarker of the neurodegenerative process; additional research is needed [73].

**Table 3:** The early optical coherence tomography (OCT) biomarkers in chloroquine/hydroxychloroquine retinopathy.

Biomarker	Description
Parafoveal thinning	The localized thinning of retinal layers in the parafoveal region detected through OCT imaging confirms the early stage of toxicity before significant visual field loss was detected [82].
Inner segment ellipsoid attenuation	The focal area of parafoveal inner segment ellipsoid attenuation with subsequent loss, particularly in the inferotemporal quadrant [83].
“Flying saucer” sign	This distinct OCT biomarker visible in the spectral domain OCT can progress from the initial stage of retinal toxicity, which is characterized by parafoveal inner segment ellipsoid attenuation [83].
“Moth eaten” photoreceptor inner/outer segments junction	This photoreceptor appearance is due to the preferential loss of cone photoreceptors, which describes the preclinical stage of hydroxychloroquine toxicity [84].

## Data Availability

Not applicable.
